# Advances in the chemical constituents, pharmacological activity, and clinical application of *Smilacis Glabrae Rhizoma*: A review and predictive analysis of quality markers (Q-markers)

**DOI:** 10.1016/j.heliyon.2024.e29557

**Published:** 2024-04-12

**Authors:** Mingxin Guo, Jiaqi Zeng, Zhanle Wang, Ying Shen

**Affiliations:** a Department of Pharmacy, The Affiliated Yixing Hospital of Jiangsu University, Yixing, 214200, China; b Pharmacology Laboratory, School of Pharmacy, Guangdong Pharmaceutical University, Guangzhou, 510006, China

**Keywords:** Chemical constituents, Pharmacological activity, Clinical application, Research progress, *Smilacis Glabrae Rhizoma* (SGR), Astilbin, Quality markers (Q-markers)

## Abstract

*Smilacis Glabrae Rhizoma* (SGR*)* is recognized in traditional Chinese medicine for its distinctive therapeutic properties and abundant supply. Its phytochemical profile is diverse, encompassing flavonoids, steroids, saccharides, phenolic glycosides, volatile constituents, organic acids, phenylpropanoids, stilbenoids, among others. Recent pharmacological investigations reveal that SGR possesses a broad spectrum of pharmacological effects with multifaceted clinical applications. This review collates the current knowledge on SGR's chemical composition, pharmacological activities, and its clinical utility. Utilizing network pharmacology and molecular docking approaches, this study provides a preliminary identification of potential quality markers (Q-Markers) within SGR. The findings suggest that compounds such as astilbin, isoengelitin, neoisoastilbin, neoastilbin, astragaloside, diosgenin, resveratrol, stigmasterol, β-sitosterol, and quercetin in SGR are promising candidates for Q-Markers. While flavonoids are the most extensively studied, there is a pressing need to further explore the active monomeric compounds within SGR. The introduction of Q-Markers is instrumental in developing standardized quality metrics. Specifically, astilbin has been noted for its antitumor, antidiabetic, antihypertensive, anti-hyperuricemic, and hepatoprotective potential, warranting further research for therapeutic applications.

## 1 Introduction

The 2020 edition of the Chinese Pharmacopoeia delineates *Smilacis Glabrae Rhizoma* (SGR) as the dried rhizome of *Smilax glabra* Roxb. from the Liliaceae family, predominantly found in regions such as Guangdong, Hunan, and Zhejiang. Historical texts, notably the Compendium of Materia Medica, distinguish between the red and white varieties of SGR, attributing higher medicinal value to the latter. SGR is reputed to bolster spleen and stomach function, enhance musculoskeletal health, and stimulate metabolic processes leading to improved circulation and muscle relaxation [1]. It has been traditionally used to alleviate pain, address gastrointestinal disturbances, and treat skin conditions, notably malignant sores and carbuncles. SGR's pharmacological profile is comprised of flavonoids, steroids, saccharides, phenolic glycosides, volatiles, organic acids, and phenylpropanoids [2], which contribute to its diverse therapeutic actions including anticancer, anti-inflammatory, antioxidant, cardiovascular, immunosuppressive, antibacterial, antiviral, uric acid reduction, and renal protection effects [3,4]. Clinically, SGR is formulated to manage urological and digestive system disorders, skin diseases, gout, and arthritis.

Despite advancements in artificial cultivation technology enhancing the availability of SGR, there is variability in its quality. The Chinese Pharmacopoeia currently prescribes astilbin content as the sole criterion for quality assessment [5]; however, this unidimensional approach is inadequate, given the complexity of SGR's constituents. The prevailing challenge is the disconnect between quality control measures and SGR's traditional therapeutic attributes, exacerbated by a dearth of foundational research and uniform quality standards. This analysis of SGR's chemical, pharmacological, and clinical profiles aims to furnish a scientific framework for its quality assessment and to propel the SGR industry towards a more robust and sustainable trajectory. In this context, we explore the Q-markers reflective of SGR's distinctive chemical makeup, clinical effectiveness, traditional benefits, and medicinal properties. Utilizing network pharmacology and molecular docking strategies, we undertake a preliminary examination of potential Q-markers within SGR. This endeavor is directed at enhancing the precision of quality evaluation and informing the future progression of SGR's therapeutic applications.

## 2 Methods

For the comprehensive elucidation of SGR's pharmacological properties, including antitumor, antioxidant, anti-inflammatory, cardiovascular protection, immunosuppression, antiviral, antibacterial activities, and its role in uric acid reduction and renal protection, we conducted a systematic literature review. Sources consulted encompassed Web of Science, PubMed, ScienceDirect, CNKI, along with an array of seminal texts in Chinese medicine, inclusive of some documents lacking verified origin. This investigation extended to the principal components of SGR, integrating the Q-markers concept to propose novel quality control benchmarks for SGR.

The literature pertaining to SGR was scrutinized, merging the identified Q-markers with data from the TCMSP database (OB ≥ 30 %, DL ≥ 0.18). Employed keywords included: SGR, chemical constituents, flavonoid, steroid, phenolic glycoside, volatile, organic acids, phenylpropanoids, stilbene, pharmacological activity, clinical application, quality marker, and research progress. Identified Q-markers were processed through Swisstargetprediction and the TCMSP database to delineate associated targets. These targets were tabulated using Microsoft Excel, facilitating the aggregation of SGR's target genes.

Subsequently, SGR target data were uploaded to the STRING database, with outputs obtained in TSV format. This information was then analyzed with the CytoNCA plugin within Cytoscape 3.9.1 software to perform network topology analysis, identify core proteins, and construct a protein interaction network. Molecular docking verified the analyzed Q-markers against key targets, with subsequent calculation of binding energies.

## 3 Results

Our investigation delineated the Q-markers of SGR by evaluating its chemical composition uniqueness, clinical efficacy, traditional medicinal effects, and properties, alongside quantifiable chemical components. Employing network pharmacology and molecular docking, we analyzed the prospective Q-markers within SGR.

### 3.1 Chemical constituents

#### 3.1.1 Flavonoids

**Fig. 1:**
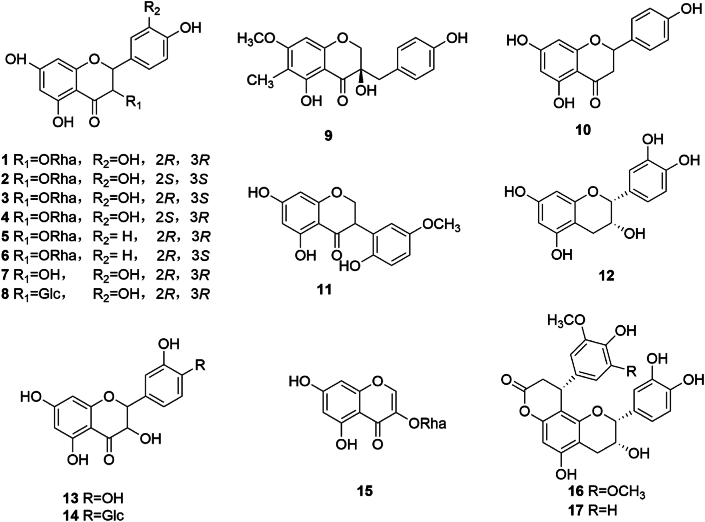
Chemical structures of flavonoids in SGR.

Flavonoids, primarily in glycoside or aglycone forms, represent the most abundant category of chemical constituents in SGR. To date, 17 flavonoids have been documented in SGR, with dihydroflavonol compounds constituting a significant portion. Notably, astilbin (**1**) [6,7], neoastilbin (**2**) [7]**,** isoastilbin (**3**) [7], neoisoastilbin (**4**) [7], astragaloside (**5**) [6,7], isoengelitin (**6**) [6], taxifolin (**7**) [6,8], taxifolin 3′*-O-*glucoside (**8**) [8], and smilachromanone (**9**) [8]. Among these, astilbin, also known as smiglabrin, is the main active ingredient of SGR, and compounds **1** to **4** are four isomers of *α*-l-mannopyranoside, which possess different absolute conformations at the 2 and 3 positions. Other flavonoids include dihydroflavonoids such as naringenin (**10**) [8]; isoflavonoids such as 7,6′-dihydroxy-3′-methoxy isoflavone (**11**); flavanols such as (−)-epicatechin (**12**) [8], smiglabrone A (**16**), and smiglabrone B (**17**) [8]; and flavonols such as quercetin (**13**) [9], quercetin-4′*-O-β*-D-glucopyranoside (**14**) [9], and 5,7-dihydroxychromanone 3*-O-α*-l-rhamnopyranose (**15**) [9], the structures of which are shown in Fig. 1.

Continued research efforts are focused on the extraction and separation of flavonoids from SGR to elucidate new molecular structures. The variability in monomer content within the 17 identified flavonoids poses a challenge in definitively determining their pharmacological profiles [10]. To date, significant pharmacological exploration has been limited to a subset of common flavonoids including astilbin, neoastilbin, isoastilbin, and neoisoastilbin. These compounds have primarily been studied for their anti-inflammatory and antioxidant activities [7,11,12].

Astilbin, the predominant bioactive constituent of SGR, has been the subject of comprehensive research, particularly for its utility in managing inflammatory pain. This research has underscored the critical role of flavonoids in both plant physiology—aiding in growth and defense against pathogens—and in pharmacological applications. Investigations into the effects of astilbin have mapped a diverse spectrum of pharmacological benefits, including cardiovascular, anti-inflammatory, immunosuppressive, hepatoprotective, renoprotective, antioxidative, antidiabetic, analgesic, anti-edema, antibacterial, and rejection-inhibitory activities, alongside its dose-dependent inhibition of casein kinase II [13].

It is posited that the anti-inflammatory and antioxidative properties attributed to SGR are linked to the presence and relative abundance of flavonoids. However, the contribution of individual flavonoids to these effects is not uniformly distributed; thus, discerning their specific roles and potential interactive effects remains critical for a thorough understanding of SGR's therapeutic potential.

#### 3.1.2 Steroids

**Fig. 2:**
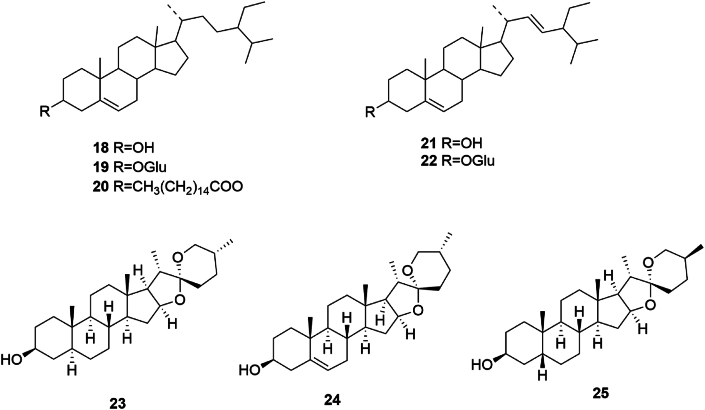
Chemical structures of steroids in SGR.

Steroids are ubiquitous in plant biology and fulfill critical roles within plant cell structures and functions. In SGR, steroids based on the perhydrocyclopentanophenanthrene nucleus have been identified. Such compounds are characterized by angular methyl groups at the C-10 and C-13 positions, a hydroxyl group at the C-3 position—often forming glycosidic linkages—and varied side chains at the C-17 position. Notable steroids from SGR include *β*-sitosterol (**18**), carotene (**19**), *β*-sitosterol palmitate (**20**), stigmasterol (**21**), stigmasterol-3*-O-β*-d-glucopyranoside (**22**), (25R)-5*α*-spirostan-3*β*-ol (**23**), diosgenin element (**24**), and (25*S*)-5*β*-spirostan-3*β*-ol (**25**) [8], the structures of which are shown in Fig. 2.

Phytosterols, a class of steroid molecules, have been recognized for their potential to modulate inflammatory responses and cholesterol metabolism. By interfering with the intestinal absorption of dietary cholesterol and enhancing metabolic degradation of existing cholesterol, phytosterols can contribute to the reduction of blood lipid levels [14]. Beyond this, phytosterols have demonstrated capabilities in the prevention of cerebral thrombosis, cancer, and ulcers; promotion of wound healing; enhancement of capillary circulation; and provision of dermatological benefits including moisturization, irritation reduction, and sun protection [15].

Within the scope of pharmaceutical interest, phytosterols, particularly *β*-sitosterols and stigmasterols from SGR, have garnered attention. The pharmacological profile of *β*-sitosterol has been associated with hypolipidemic, anti-inflammatory, and anticancer activities. Stigmasterols, in addition to their antioxidant and anti-inflammatory effects, have shown promise in antitumor activity, cholesterol reduction, and cognitive enhancement [[16], [17]].

Despite these insights, comprehensive research on the sterols present in SGR remains limited. Future investigations are warranted to ascertain the full pharmacological potential of these compounds and to verify whether their activity substantiates the efficacy attributed to SGR.

#### 3.1.3 Phenolic glycosides

**Fig. 3:**
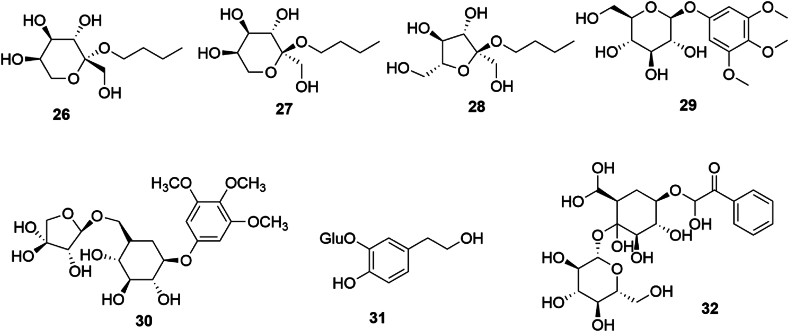
Chemical structures of phenolic glycosides in *SGR*.

Phenolic glycosides are characterized by their formation through the condensation of phenolic hydroxyl groups and sugar molecules, predominantly pyranose glucosides. In SGR, these compounds include *n*-butyl-*β*-d-fructopyranoside (**26**), n-butyl-*α*-d-fructopyranoside (**27**), n-butyl-*α*-d-furanoside (**28**), 3,4,5-trimethoxyphenyl-1*-O-β*-d-glucopyranoside (**29**), 3,4,5-trimethoxyphenyl-1*-O-*[*β*-d-furanosyl-(16)]-*β*-d-glucopyranoside glucoside (**30**), 3,4-dihydroxyphenylethanol-3*-O-β*-d-glucopyranoside (**31**), and 2,4,6-trihydroxyacetophenone-2,4-di*-O-β*-d-glucopyranoside (**32**) [18,19], the structures of which are shown in Fig. 3.

Recent investigations have contrasted the hexose and starch fractions in plants, examining polysaccharides commonly found in therapeutic flora. These large biomolecules are typically categorized into intracellular forms such as starch and extracellular forms like cellulose. Their structural composition, efficacy, and spectrum of biological activities often fluctuate with the plant species, environmental conditions, and climatic variations [20].

SGR's distinctive sweet taste is attributed primarily to its phenolic glycoside content. Consumption of SGR, which can serve as both a food and a therapeutic agent, typically involves steaming, resulting in the dissolution of phenolic glycosides into the liquid. Nonetheless, the theoretical framework to fully explain SGR's efficacy remains to be elucidated. Plant polysaccharides, including those found in SGR [14], have been linked to a range of pharmacological effects such as anticancer, antioxidant, hypoglycemic, and lipid-lowering properties [21]. Current research on SGR's polysaccharides has concentrated on their immunomodulatory impact, reflecting a significant area of scientific inquiry [22].

#### 3.1.4 Volatiles

**Fig. 4:**
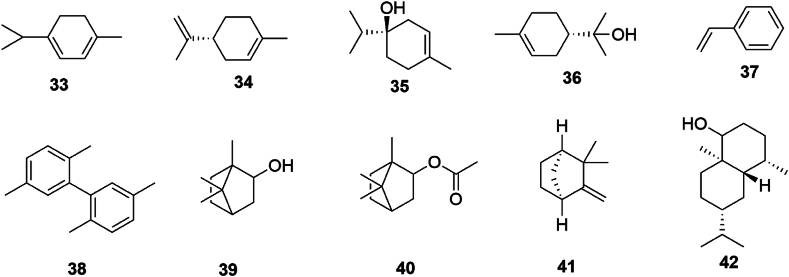
Chemical structures of volatile compounds in *SGR*.

SGR is notable for its abundance of volatile constituents, exhibiting a rich diversity. Through organic solvent-water distillation of SGR, a variety of volatile elements have been isolated, primarily consisting of terpenoids, aliphatic and aromatic hydrocarbons, and alkanes. The specific volatile compounds identified include terpinene (**33**), (+)-limonene (**34**), (−)-4-terpinol (**35**), (−)-terpineol (**36**), styrene (**37**), 2,2′,5,5′-tetramethylbiphenyl (**38**), l-borneol (**39**), bornyl acetate (**40**), camphene (**41**), *β*-eudesmol (**42**), acetaldehyde, n-octanal, decanal, (*E*,*E*)-2,4-decadienal, lauric aldehyde, hexadecyl ether, myristic acid, palmitic acid, n-nonane, n-nonane, tridecane, hexadecane, 7-methyl-octadecane, 2,6,10,14-tetramethyl-octadecane, docosane, eicosane, and tridecane [18]. The chemical structures of compounds **33** to **42** are shown in Fig. 4.

Despite the isolation and characterization of numerous volatile analogs from SGR, their pharmacological activities have not been extensively explored. Investigations into SGR volatiles have largely been directed towards refining the extraction processes of these compounds. Further research is imperative to ascertain whether these volatiles contribute to SGR's distinct sweet flavor profile. The hypothesis that volatile analogs from SGR offer negligible therapeutic benefits necessitates empirical validation.

#### 3.1.5 Organic acids

**Fig. 5:**
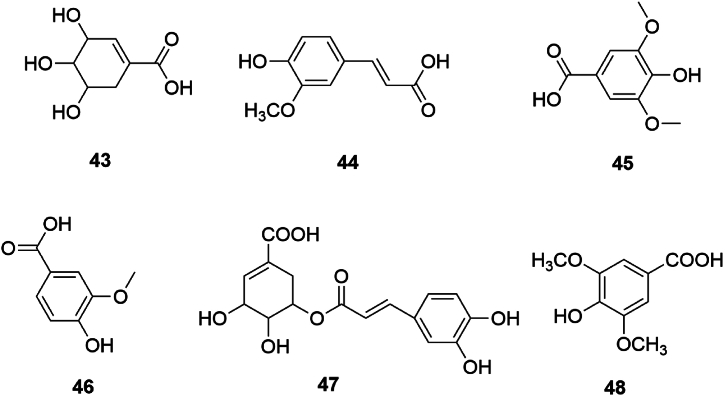
Chemical structures of organic acids in SGR.

SGR comprises various organic acids, predominantly small molecules enriched with hydroxyl, carboxyl, and other acidic functionalities. These include shikimic acid (43), ferulic acid (44), syringic acid (45), vanillic acid (46), 5-O-caffeoyl shikimic acid (47), butyric acid (48), and others such as succinic acid and palmitic acid [7,23]. Compounds 43 to 48 are delineated in Fig. 5.

Research on SGR's organic acid content has revealed that succinic acid contributes to antispasmodic, mucolytic, and diuretic effects [14]. However, the corpus of research on these organic acids is not exhaustive; while certain structures have been isolated and identified, analytical data on these organic acids is scant. Predominantly present in the rhizome of SGR, further investigations into these organic acids should prioritize this part of the plant. Chen et al. have identified succinic acid and palmitic acid as prevalent fatty acids in SGR [24]. Wang et al. have reported the efficacy of succinic acid in combatting epilepsy and palmitic acid's role in apoptosis via endoplasmic reticulum stress, potentially triggering disease [25]. The presence of palmitic acid in SGR may pose risks of adverse effects, yet no reports substantiate this concern, which could relate to its concentration or interaction with other components modulating its toxicity.

#### 3.1.6 Phenylpropanoids

**Fig. 6:**
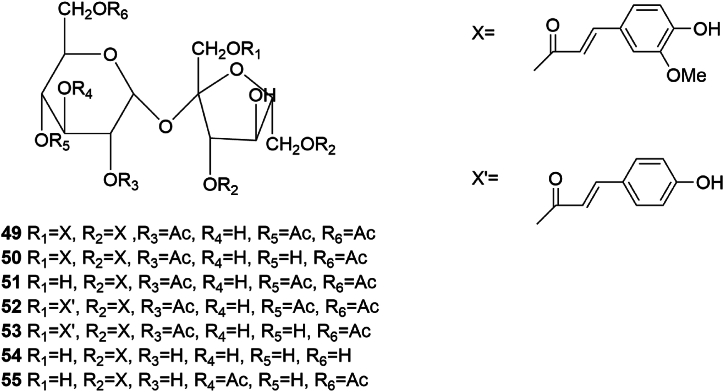
Chemical structures of phenylpropanoids in SGR.

Few phenylpropanoids have been isolated from SGR; those that have been identified mainly include five porin glycosides A (**49**)/B (**50**)/C (**51**)/D (**52**)/E (**53**), and helonioside A (**54**) and (3,6-di*-O-*feruloyl)-*β*-d-fructofuranosyl-(3,6-di*-O-*acetyl)-*α*-d-glucopyranoside(**55**) [26]. The structures of compounds **49** to **55** are shown in Fig. 6.

Recent research indicates that phenylpropanoids exhibit diverse biological activities, such as anticancer, anti-inflammatory, antiplatelet, insecticidal, antibacterial, and antiviral properties [27]. Despite the limited diversity and quantity of these compounds in SGR, their known therapeutic attributes—including tumor inhibition, antioxidation, antibacterial, antiviral activities, and cardiovascular protection—do not permit definitive conclusions regarding their role in SGR's pharmacological profile. Nevertheless, phenylpropanoids hold potential as foundational elements for pharmacological agents.

#### 3.1.7 Stilbenes

**Fig. 7:**
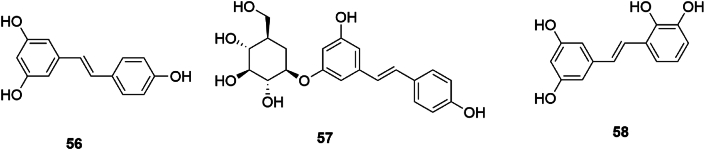
Chemical structures of stilbenoids in SGR.

Stilbenes encompass a class of compounds characterized by a homodiene styrene core structure or their polymeric derivatives. Isolated stilbenes from SGR include resveratrol (56), resveratrol-3-O-*β*-D-glucopyranoside (57), and smiglastilbene (58) [23,28], with the molecular conformations depicted in Fig. 7.

Stilbenes, commonly located in plant xylem, demonstrate a broad spectrum of biological functions: antioxidative, antiviral, antineoplastic, anti-inflammatory, and hypoglycemic actions, as well as modulation of insulin sensitivity and lipid metabolism [29]. Resveratrol, a naturally occurring stilbene, exhibits antioxidative, anti-aging, and glucose-lowering effects, and has been associated with reduced cardiovascular disease risk [30]. Glycosylated derivatives of resveratrol engage the casein kinase 2 interacting protein 1-nuclear factor E2-related factor 2-antioxidant response element signaling pathway, potentially mediating antidiabetic nephropathy [31]. While stilbenes present a promising foundation for antihyperglycemic pharmacotherapy, current research does not substantiate a marked hypoglycemic effect from SGR, suggesting that these compounds may contribute to other therapeutic benefits or that their efficacy is not pronounced.

#### 3.1.8 Other components

SGR extracts have yielded alkaloids, tannins, and heterocyclic compounds. Alkaloid content in SGR rhizomes was measured at 0.0349 %, and tannins were quantified at 20.177 mg/g in the plant matrix [32]. Proteinaceous substances, including a heterodimeric non-mannose-binding hemagglutinin (MW: 32 kDa), a trimeric mannose-binding hemagglutinin (MW: 37 kDa), and a protein with a sequenced N-terminus (MW: 30 kDa), have also been identified. Sugars such as glucose, fructose, and starch, along with inorganic elements like Ca, Fe, Mg, Cd, Mn, Zn, and Cu, were analyzed via flame atomic absorption spectrophotometry [7,23]. Given the complexity and diversity of compounds in Chinese herbal medicines, ascertaining the material basis for their medicinal effects from singular or multiple compounds is challenging. Consequently, each constituent's role in the comprehensive medicinal impact of such botanical drugs merits further investigation.

### 3.2 Pharmacological activities

#### 3.2.1 Antitumor activity

SGR's detoxifying and dispersing attributes contribute to its emerging anticancer properties. Extracts and individual compounds from SGR have demonstrated efficacy in impeding the proliferation and migration of various cancer cell lines. Research indicates that SGR exhibits a broad-spectrum antitumor capability, suppressing growth and invasion of gastric cancer cells (SGC7901) [26], prostate cancer cells (LNCaP) [33], and liver cancer cells (HepG2 and Hep3B) [34]. These effects may be effected through the inhibition of Akt phosphorylation at Thr308, downregulation of *β*1 integrin expression, induction of cell cycle arrest at the S or S/G2 phase transition, and enhancement of apoptosis. Furthermore, a dose-dependent decrease in migration and adhesion of tumor cells has been observed. Gao et al. demonstrated that SGR extract inhibited the proliferation and migration of human breast cancer cells (MCF7), colon cancer cells (HT-29), and gastric cancer cells (BGC-823) in a dose-dependent manner, suggesting mitochondrial mediation of apoptosis as a mechanism of anticancer action [35]. Additionally, SGR showed tumor growth inhibition in HT-29-xenografted Balb/c nude mice and murine hepatoma H22-bearing mice. The antitumor activity of SGR ethyl acetate extract may involve the suppression of the HIF-1 signaling pathway and modulation of tumor-associated macrophages towards the M1 phenotype, enhancing antitumor immune response.

Flavonoids constitute the principal antitumor constituents of SGR. Research by Jiang et al. revealed that total flavonoids extracted from SGR ameliorated renal injury in Lewis lung carcinoma-bearing mice undergoing cisplatin chemotherapy, attributed to upregulation of Nrf2 expression. Furthermore, the flavonoid astilbin was shown to attenuate cisplatin-induced apoptosis in human renal tubular epithelial cells (HK-2) by downregulating OCT2 protein expression. Astilbin also augmented the pro-apoptotic effect of cisplatin on human lung cancer cells (A549) via the Bax/Bcl-2/caspase-3 pathway [36,37]. SGR's ethyl acetate fraction has been implicated in antitumor activities, notably by inhibiting the HIF-1 signaling pathway and reprogramming tumor-associated macrophages towards an M1 phenotype [38]. Additionally, saponins in SGR hold promise as raw materials for developing antitumor functional foods, demonstrating marked anticancer efficacy. Selective inhibition of sarcoma S180 cells by SGR saponins has been confirmed both in vitro and in vivo [39].

The therapeutic target organ selection for SGR correlates with its recognized meridian affinities. Traditional Chinese Medicine (TCM) posits that the medicine's effects are meridian-specific, influencing particular Zang-fu organs. The predominant documentation, including the Chinese Pharmacopoeia, aligns SGR with the liver and stomach meridians, whereas some sources attribute it to the kidney and large intestine meridians. This does not confine SGR's anticancer properties but suggests that its molecular targets for other medicinal benefits may be primarily associated with the liver and stomach and potentially extend to the kidney and large intestine. In TCM context, 'organ' refers to systems rather than discrete anatomical structures; therefore, SGR's target organs might be situated within the digestive and metabolic systems. Current empirical studies on SGR's antitumor effects primarily address liver and stomach pathologies, yet there is potential for its therapeutic application to extend to neoplasms of other organs.

#### 3.2.2 Antioxidant and anti-inflammatory activities

The antioxidant and anti-inflammatory properties of SGR's flavonoids have been substantiated through pharmacological research. These compounds have been demonstrated to mitigate renal oxidative stress and inflammation, and they possess therapeutic potential for treating rat models of rheumatoid arthritis induced by complete Freund's adjuvant [3,40]. Analytical studies have identified (−)-epicatechin, astilbin, neoastilbin, and isoastilbin as active agents within SGR, exhibiting potent antioxidant capabilities in DPPH and ABTS + assays, along with the ferric reducing antioxidant power (FRAP) system [41]. These constituents also reduce the expression of pro-inflammatory cytokines IL-1*β*, IL-6, and mediators NO, and NF-κB p-p65 in lipopolysaccharide-induced RAW 264.7 cells [42]. Fucoidan's antioxidant activity has been attributed to the modulation of ERK1/2, c-JNK, and p65 pathways and the activation of the Keap1/Nrf2/HO-1 axis. Concurrently, its anti-inflammatory action involves the suppression of ROS, NO, and TNF-α synthesis, coupled with inhibition of the NF-κB signaling pathway [43]. Additionally, polysaccharides from SGR have been recognized for their anti-inflammatory efficacy [44].

Lu et al. documented that phenolic-enriched extracts from SGR are capable of neutralizing free radicals and reducing TNF-α, IL-6, and NO levels, thus serving both antioxidant and anti-inflammatory functions [45]. The ethanolic extracts from SGR leaves and seeds have demonstrated significant antioxidant activity, offering protective effects against UV-induced skin aging in skin fibroblasts [46]. While phytosterols in porphyra have been proposed as key anti-inflammatory and analgesic agents, empirical evidence from isolated studies remains scarce. Nevertheless, anti-inflammatory and analgesic properties are frequently associated with SGR. Xue et al. posited that phytosterols are pivotal constituents within SGR, conferring anti-inflammatory benefits and promoting wound healing [14].

In conclusion, flavonoids and phenols are the foundational elements for SGR's antioxidant activities. Ethanol extracts from the plant's leaves and seeds present potential for antiaging pharmacological development, indicating possible expansion in clinical applications. The extraction and purification of SGR's chemical components, particularly from the rhizome, warrant further investigation. Moreover, comprehensive medicinal properties of other parts of the plant merit consideration. The human anti-inflammatory efficacy of phytosterols requires confirmation through future clinical research.

#### 3.2.3 Protection of the cardiovascular system

The rising incidence of cardiovascular diseases, exacerbated by economic growth and increased living standards, now poses a significant risk to human mortality. Studies on SGR have indicated its efficacy in the prevention and treatment of cardiovascular ailments, specifically through the reversal of cardiac hypertrophy and the reduction of glycemia and hypertension. Glycosides and flavonoids in SGR have been the focal points of these investigations.

Cai et al. observed that flavonoid extracts from SGR could attenuate Ang II-induced cardiac hypertrophy at concentrations of 0.5–1.0 mg/mL. This effect may be associated with the inhibition of RyR-mediated intracellular Ca2+ release and the subsequent upregulation of JP2 and RyR2 mRNA and protein expression in cardiomyocytes [47]. Furthermore, SGR has demonstrated a significant decrease in systolic, diastolic, and mean arterial pressures in models of renal hypertension. It also reduced atrial natriuretic peptide (ANP) and endothelin (ET) levels while increasing nitric oxide (NO) concentrations, contributing to its antihypertrophic and antihypertensive properties.

Ethyl acetate extracts of SGR have prevented epinephrine-induced arrhythmias in rabbits, exhibiting sedative effects. These extracts have elicited *β*-blocker-like actions akin to propranolol, diminishing ANP and ET levels and elevating NO, thereby producing hypotensive effects [48]. Smiglabrin, a component of SGR, was shown to inhibit the velocity and amplitude of slow-response action potentials in cardiac cells post-isoprenaline administration under hyperkalemic conditions. This suggests an inhibitory action on receptor-controlled channels activated by isoprenaline, with no significant impact on the voltage-dependent channels activated by calcium chloride. In comparative studies, high doses of smiglabrin outperformed ligustrazine, a standard anti-atherosclerotic agent.

Provided the safety profile of SGR can be established and an effective dosage range determined, the prospects of developing functional foods for the middle-aged and elderly populations are promising. Such products would leverage the cardioprotective, glycemic, and antihypertensive effects of SGR's bioactive compounds, especially its flavonoids and saponins, which offer substantial preventive benefits against cardiovascular diseases.

#### 3.2.4 Immunomodulatory properties

The immunomodulatory attributes of SGR are evidenced by its active component astilbin, a flavonoid known for potent immune modulation. Investigations have revealed that astilbin suppresses NO secretion, downregulates inducible NO synthase expression in murine models, and, in conjunction with lipopolysaccharide (LPS), instigates the activation of signaling pathways involving phosphorylated P65-ERK1/2 and JNK proteins. These pathways contribute to the enhanced immunomodulatory function of macrophages.

Zou et al. demonstrated that astilbin significantly impedes the maturation of dendritic cells (DCs) and curtails their antigen-presenting capacity, which in turn diminishes T cell proliferation and activation within a BALB/c murine heart transplant model [49]. This finding posits astilbin as a viable candidate for immunosuppressive intervention following cardiac implantation. Moreover, the dualistic influence of astilbin on immune cells is noted, as it both inhibits DC maturation and fosters macrophage immune activity when combined with LPS. Additionally, lysine has been shown to inhibit the maturation of bone marrow-derived DCs in a dose-responsive manner during in vitro studies, consequently downregulating immune function [50].

The immunostimulatory effects of SGR appear to be associated with the upregulation of CD4+T cells and the attenuation of the pro-inflammatory cytokine IFN-α. Further evidence suggests that SGR's aqueous extract increases the populations of CD3^+^ and CD4^+^ splenic T cells, thereby bolstering immune functionality. CD4+T cells are instrumental in driving lymphocyte differentiation and antibody production, which are critical for immune response [51]. Wang et al. observed that the SGR aqueous extract selectively inhibits inflammation by blocking the release of lymphokines from sensitized T lymphocytes, and uniquely suppresses cellular but not humoral immune responses [2].

The antiallergic properties of SGR are pronounced in its ethanol extract (concentrations of 95 % and 50 %), whereas its aqueous extract exhibits minimal antiallergic activity (IC50 > 100 μmol/L). The antiallergic potential of SGR is likely attributable to the synergy of bioactive compounds within its crude extract, warranting further investigation to identify the optimal extraction solvent to enhance this activity. SGRP1, a heteropolysaccharide fraction from SGR, has been identified as another compound with confirmed immunomodulatory impact on RAW 264.7 macrophages. Current research points to flavonoids and phytopolysaccharides as the primary agents of SGR's immunomodulation. Although the immunoregulatory effects of SGR's aqueous extract may predominantly arise from flavonoids and phytopolysaccharides, it is important to recognize that other lipophilic constituents within SGR may also influence immune processes, since both aqueous and ethanolic extracts are capable of modulating immune responses, and the solubility of flavonoids and phytopolysaccharides is primarily in water. The SGR aqueous extract has been clinically applied in the treatment of cellular immunological disorders.

#### 3.2.5 Antibacterial and antiviral activity

The antibacterial properties of SGR encompass a broad spectrum, displaying potent activity against both Gram-positive and Gram-negative bacteria. Empirical studies have confirmed that extracts from SGR exhibit significant inhibition rates against a variety of pathogens, including *Listeria monocytogenes*, *Escherichia coli*, *Salmonella enteritidis*, *Staphylococcus aureus*, *Endomyces albicans*, *S. falciparum*, *Bacillus diphtheria*, and *Bacillus anthracis*. Conversely, a marginally reduced antibacterial effect is observed against certain strains such as *E. coli*, *hemolytic streptococcus*, *Pseudomonas aeruginosa*, and *Salmonella typhimurium* [52]. Notably, the antibacterial efficacy against *E. coli* is not affected by the pathogen's production of ESBLs, suggesting an advantage of SGR over conventional antibiotics in curbing the proliferation of drug-resistant bacterial strains [53]. However, the precise mechanisms underlying the antibacterial action against *E. coli* remain inadequately explored, and the active component responsible for this effect is yet to be identified.

Regarding antiviral capabilities, glycoproteins derived from SGR have demonstrated activities against respiratory syncytial virus (RSV) and herpes simplex virus type 1 (HSV-1) [54]. Clinical observations by He et al. indicate successful applications of Chinese herbal remedies incorporating SGR in the integrative treatment of novel coronavirus pneumonia [55]. Furthermore, several clinical reports have documented favorable outcomes using SGR-based formulations in managing hepatitis B virus-related slow plus acute liver failure (ACLF) [56], highlighting the presence of antiviral constituents within SGR. While these findings underscore the potential for SGR-derived compounds in antiviral drug development, the specific active ingredients and their mechanisms of action remain to be elucidated. Wang et al. propose that the antibacterial properties of Chinese herbal medicine, which SGR is a part of, may be linked to the abundance of hydroxyl groups in their constituents, which are capable of resonating at frequencies that coincide with the vibrational wavelength of bacteria and viruses, thus facilitating their elimination [2].

The antimicrobial properties of SGR appear to stem from the synergistic effects of various bioactive constituents. Evidence suggests a notable association with glycosides, such as astilbin, isoengeletin, daucosterol, and saponin; organic acids, including succinic acid, palmitic acid, ferulic acid, and shikimic acid; as well as tannins and resins. The efficacy of these compounds in aqueous extracts of SGR points to their substantial role in the extract's antimicrobial activity. Current research implicates flavonoids, alkaloids, and tannic acid in SGR as key agents conferring antimicrobial benefits. When formulating antimicrobial and antiviral agents incorporating SGR, it is imperative to optimize the preparation method to prevent the degradation or loss of these essential components, particularly given the difficulty in pinpointing the exact molecules at play. Moreover, despite promising in vitro studies, the therapeutic potential of SGR-derived flavonoids, alkaloids, and tannic acid requires validation through extensive clinical trials to establish their efficacy in human treatments.

#### 3.2.6 Uric acid reduction and renal protection

Hyperuricemia, a prevalent metabolic disorder, arises primarily due to anomalies in purine metabolism and compromised uric acid excretion [57]. Xanthine oxidase (XOD), pivotal in uric acid synthesis, facilitates the conversion of xanthine and hypoxanthine to uric acid. Inhibitors targeting XOD activity can mitigate uric acid synthesis, and several clinical hyperuricemia therapeutics include SGR derivatives.

Research has identified several SGR constituents, such as Douglas-fir-3-O-α-L-rhamnopyranosyl-α-L-rhamnopyranoside, engeletin, 4-(pyrrolo[3,2-D]pyrimidin-4-yl)phenol, and resveratrol, as XOD inhibitors, thereby implicating them in uric acid reduction. Notably, flavonoids from SGR have garnered attention for their uric acid-lowering capabilities. Among these, 4-(pyrrolo[3,2-D]pyrimidin-4-yl)phenol exhibits potent XOD inhibitory action but is not abundantly present in SGR. Engeletin and isoengeletin are hypothesized to be the primary active components that contribute to the reduction of uric acid levels in SGR [58].

The mechanism by which SGR inhibits XOD may involve multiple pathways: (1) suppression of urate anion exchanger 1 (URAT1) mRNA expression; (2) modulation of uric acid reabsorption and secretion via URAT1; (3) reduction of IL-1 and TNF-α expression in vascular endothelial cells, thereby attenuating the impact of uric acid on these cells; and (4) upregulation of uric acid transporter proteins like CLUT9 and the ATP-binding cassette sub-family G member 2 (ABCG2).

SGR extends beyond uric acid reduction to afford renal protection [59]. Isolated flavonoids from SGR have demonstrated the ability to suppress XOD activity and enhance the expression of organic anion transporter 1 (OAT1) and organic cation/carnitine transporter 2 (OCTN2) in renal tissue, exerting anti-epithelial-mesenchymal transition (EMT) and mitigating renal interstitial fibrosis (RIF). These effects may occur through the inhibition of the TGF-*β*1/SmaD signaling pathway and its downstream targets, including the miR-21/PTEN axis [60].

Astilbin, a component of SGR extract, has been shown to curtail the production of TGF-*β*1 and connective tissue growth factor (CTGF) stimulated by high glucose levels in HK-2 cells. It is also effective in ameliorating renal function, decreasing renal indices, and offering protection against experimental diabetic nephropathy in both in vitro and in vivo settings, thus serving as a potential therapeutic against diabetic nephropathy [13].

Advanced glycation end-products (AGEs) have been implicated as primary contributors to diabetic vascular complications. Flavonoids, the most abundant and readily extractable constituents of SGR, can counteract AGE-induced endothelial dysfunction, notably through the RAGE-ERK1/2-NF-κB signaling pathway. While the role of flavonoids has been extensively studied, research on stilbenes, despite demonstrating antidiabetic properties, remains scant, particularly in relation to their pharmacological significance in SGR.

Empirical pharmacological data suggest SGR's affinity for the liver meridian and possibly the kidney meridian, highlighting its prospective utility in treating metabolic disorders, given that the kidneys are vital for metabolic processing and excretion. The potential of SGR, especially its stilbene components, in managing metabolic diseases warrants further investigation, considering the possibility of yet unidentified factors influencing their pharmacological activity.

#### 3.2.7 Other effects

In addition to its established pharmacological properties, SGR exhibits a spectrum of other therapeutic effects including hepatoprotection, anti-gastric ulcer, and headache relief. Notably, SGR flavonoids have reversed carbon tetrachloride (CCl4)-induced hepatic damage in rodent models, indicating hepatoprotective capabilities. These flavonoids also promote the formation of small lipid droplets and decrease intracellular free fatty acid levels in mice, suggesting a preventive role against obesity and associated metabolic conditions [61,62].

SGR aqueous extract has demonstrated a protective role in acute liver injury induced by carbon tetrachloride through the inhibition of the NLRP3 inflammasome activation [63]. Furthermore, Smilax has been observed to offer dose-dependent protection against water immersion stress-induced gastric ulcers in mice [64].

Network pharmacological analyses have identified naringenin, *β*-sitosterol, quercetin, diosgenin, and stigmasterol as the primary active constituents of SGR for treating fever [58]. Herbal formulations incorporating SGR have shown efficacy in promoting the excretion of heavy metals like lead and mercury and have countered oxidative liver damage in lead-induced toxicity in mice [65,66].

The pinpointing of specific pharmacological actions within plant-based therapies is challenged by their complex and selective targeting nature. Additionally, despite advances in extraction, separation, and identification techniques, the monomers isolated do not always equate to the full potency of the plant. The efficacy of a botanical drug may vary with changes in the ratio of active component concentrations and the physiological state of the organism.

Thus, it is imperative to establish varied conditions for confirming the pharmacological activities of SGR. Table 1 encapsulates a summary of the pharmacological activities and the pharmacodynamic material basis of SGR.

**Table 1:** Pharmacological activities of SGR.

Pharmacological activities	Pharmacological Models	Pharmacological effect	Dosage	Pharmacodynamic constituents/	References
Antitumor activity	SGC7901	Inhibiting the proliferation and invasion and enhance its apoptosis through Akt-mediated signaling pathways.	10, 20, 30 mmol/L	SGR extract	[26]
	LNCaP	Reducing the expression of the 1 integrin, which is collagen-dependent, in LNCaP cells.	50–200 μg/mL		[33]
	MCF7、HT-29、BGC-823	Through mitochondrial regulation of apoptosis.	0–100 mg/mL		[35]
	Lewis lung cancer mice	Promoting Nrf2 expression	300 mg/kg	Total flavonoids of SGR	[36]
	HK-2	Inhibiting OCT2 protein expression	30, 50, 100, 200, 300 μM	Astilbin	[37]
	A549	Regulating Bax/Bcl-2/caspase-3 apoptotic pathway	2 mg/L	SGR extract	[38]
Antioxidant and anti-inflammatory activities	Rheumatoid arthritis in rats induced by full Freund's adjuvant	Up-regulation of ERK1/2, JNK, and p65 phosphorylation and activation of the Keap1/Nrf2/HO-1 pathway.	100, 300, 500 mg/kg		[40]
	lipopolysaccharide (LPS)-induced RAW 264.7 cells;	Inhibition of ROS, NO, and TNF-*α* production and the NF-κB pathway.	15.05 mg/kg, 12.5–50 mg/mL	Astilbin; Phenolic-Enriched Extract;	[42]
	HS68	Decreased Ca^2+^ and ROS, mitochondrial membrane potential, dysfunction, and increased glutathione, NADPH dehy drogenase and heme oxygenase-1	50, 100, 200 μg/mL	Ethanolic extract	[46]
Protection of the cardiovascular system	Wistar rats	Inhibition of RyR-mediated intracellular Ca2+ release and upregulation of JP2, RyR2 mRNA and protein levels; Reduce blood pressure ANP and endothelin ET; Increase the levels of NO; Anti-cardiac hypertrophy; Blood pressure-regulating;	0.5–1.0 mg/mL	Flavonoids	[47]
	SD rats	Reduce ANP and ET; Elevate NO levels;	1.5, 3, 6 g/kg	Ethyl acetate extract of SGR	[48]
	C57BL/6 mice	Suppressing T cell proliferation and activation	75, 125, and 250 mg/kg/d	Astilbin	[49]
	BALB/c mice	Inhibit the maturation of bone marrow-derived DCs; Negatively modulate their immune function;	25 μg/mL, 50 μg/mL, 100 μg/mL	Lysine	[50]
Antibacterial and antiviral activity	Reference strains and clinical isolates	Compelling antibacterial activity and a high inhibition rate against *Listeria monocytogenes, Escherichia coli, Salmonella enteritidis,* *Staphylococcus aureus, Endomyces albicans, S. falciparum, Bacillus diphtheria, and Bacillus anthracis;* A slightly weaker antibacterial activity against *E. coli, hemolytic streptococcus, Pseudomonas aeruginosa, and Salmonella typhimurium*	–	SGR extract	[52]
	*Escherichia coli*.	It has an advantage over traditional antibiotics in inhibiting the growth of drug-resistant bacteria	0.39–100 mg/mL	*Smilax glabra* Roxb. granules	[53]
	RAW264.7 cell line	Antiviral andantiproliferative activities against RSV and HSV-1	–	Glycoproteins	[54]
	patients	Good efficacy for treating COVID-19	–	Chinese herbal prescriptions containing SGR	[55]
	ACLF patients	Increase the improvement rate; Improve the clinical symptoms and liver function; Raise the 6 months survival rats;	≥100g	SGR composition	[56]
Uric acid reduction and renal protection	Rats or patients	Inhibit the activity of XOD; Lower uric acid;	37.5, 18.75 mg/kg	Resveratrol; Douglas-fir-3-O-*α*-L-rhamnopyranosyl-*α*-L-rhamnopyranoside, engeletin; 4-(pyrrolo[3,2-D]pyrimidin-4-yl)pheno	[57,58]
	ICR mice	Down-regulation of serum uric acid and creatinine and up-regulation of albumin and hematocrit levels	10 g/kg	SGR	[59]
	Male Kunming mice	Inhibit XOD activity; Upregulate the expression of OAT1 and OCTN2;	250, 125, and 62.5 mg/kg;	Flavonoids	[60]
	Rats	Anti-epithelial-mesenchymal transition (EMT) and renal interstitial fibrosis (RIF) effects.	60, 30 and 15 mg/kg;	Flavonoids	[60]
	HK-2 cells; SD rats;	Improve renal function, reduce renal index, and confer protective effects against experimental diabetic nephropathy.	0.3, 1, 3 μM	Astilbin	[13]
Other effects	Male Sprague-Dawley rats	Reduced the elevated activities of ALT and AST; Reverse CCl4-induced hepatic injury;	100, 300 and 500 mg/kg	Flavonoids	[62]
	Mouse 3T3-L1 cells	Increase the phosphorylation of AMPK; Decrease the phosphorylation of AKT in adipocytes; Reducing intracellular free fatty acid levels; Prevent obesity and related metabolic disease	62.5, 125, and 250 μg/mL	Flavonoids	[61]
	C57BL/6	Dose-dependently protected against water immersion stress gastric ulcers.	100, 200, 300 mg/kg	SGR	[64]
	–	Regulate the body's response to external stimuli and resistance to EGFR inhibitors. --Treat fever	–	Naringenin; *β*-sitosterol; Quercetin; diosgenin; Stigmasterol;	[58]
	ICR mice	Decrease rate of MDA levels in liver; Increase rate of GSH levels and the activities of GSH-PX and SOD in liver; --significantly advance the excretion of lead and protect liver against oxidative damage induce by lead.	2, 5, 10 g/kg	Traditional Chinese herbal formula with SGR	[65]
		--significantly advance the excretion of mercury			[66]

### 3.3 Clinical applications

#### 3.3.1 Treatment of urinary system diseases

Urinary system disorders, characterized in Traditional Chinese Medicine as "stranguria with turbid discharge," are often attributed to damp-heat, blood stasis, and renal insufficiency. SGR has demonstrated effective detoxification and anti-rheumatic properties [67]. The utilization of a dampness-eliminating formula, incorporating SGR alongside Tsubaki Root, White Bark, Celosia, Coicis Semen, and White Peony, has yielded positive outcomes in treating female leucorrhea abnormalities [68]. The primary objective in managing urinary system diseases is the abatement of inflammation, and formulas containing SGR have been efficacious in swiftly mitigating symptoms of urinary urgency, frequency, and pain in acute urinary tract infections, typically within 1–2 days.

Sun et al. have reported a 97.12 % effectiveness in treating non-gonococcal urethritis using a combined approach of Western and Traditional Chinese Medicine, specifically Tonglin Detoxification Tang, which includes SGR and other herbs. This was significantly superior to the 85.58 % effectiveness with Western medicine alone [69]. Additionally, the adjunct use of microwave therapy with Qingzhuo Tang—a blend of SGR, Peach Kernel, Safflower, Red Peony, and Salvia—has notably decreased the National Institutes of Health Chronic Prostatitis Symptom Index (NIH–CPSI) scores, enhanced quality of life, increased the count of lecithin vesicles, and lowered recurrence rates in chronic non-gonococcal prostatitis [70].

SGR's role as a principal agent in treating gonorrhea, urinary tract infections, and recalcitrant fungal vaginitis has also been corroborated with remarkable efficacy.

#### 3.3.2 Treatment of digestive system diseases

SGR, a widely used Traditional Chinese Medicine (TCM), has shown greater efficacy in fostering the healing of gastric ulcers and ameliorating chronic gastritis inflammation when compared to Western medicinal treatments. The combined application of cimetidine, SGR, and Notoginseng in a hemostatic decoction has been effective in halting hemorrhage, preventing recurrent bleeding, and facilitating peptic ulcer healing [23]. Additionally, the Weikang Mixture, containing SGR, Sanguis Draxonis, White Peony, and Cyperus rotundus L., has outperformed conventional Western treatments in improving gastric mucosa symptoms and inflammation in chronic gastritis cases.

Herbal medications, including an antibacterial concoction with SGR, Salvia, Forged corrugated, and Dai Ochre, have achieved a 92.5 % success rate in treating Helicobacter pylori-induced gastric ulcers, surpassing the 75 % efficacy rate of Western medications [71]. Moreover, formulas designed to clear heat, dispel dampness, and detoxify—comprising SGR, Angelica, Coptis chinensis Franch., Jiao Hawthorn, and Jiao medicated leaven—have demonstrated a capacity to diminish or eliminate damage to the colonic mucosa and offer preventive and therapeutic benefits against colitis Gravis [72].

#### 3.3.3 Treatment of skin diseases

In TCM, SGR is reputed for its ability to target and alleviate damp-heat and toxins within the body, making it a common choice for treating skin conditions such as psoriasis and hand-foot-and-mouth disease [73]. TCM formulas like the Jiawei Xiaoyin decoction with SGR, *Bubali Cornu*, Dried Rehmannia Root, *Paeoniae Radix Rubra*, and Ramuli Euonymi, and the Indigo Naturalis decoction with SGR, Indigo Naturalis, *Lonicerae Japonicae Flos,* Glycyrrhizae Radix Et Rhizoma, and Smilax China have significantly lowered TNF-α and VEGF levels in patients with psoriasis. These treatments have been effective in rapidly reducing symptoms and are considered to have fewer side effects, warranting further clinical promotion [74].

Furthermore, treatments like the spleen-invigorating and skin-beautifying powder, incorporating SGR, Astragalus mongholicus, *Polygonati Rhizoma*, and *Atractylodis Rhizoma*, have demonstrated superiority over oral antiallergy medications in managing infantile eczema associated with spleen deficiency and damp accumulation, as assessed by EASI scores, TCM systemic evidence scores, and overall efficacy [75,76]. The Wang Huo Huang lotion, which includes SGR, *Rhei Radix Et Rhizoma*, Pogostemonis Herba, Golden Larch Bark, and *Phellodendri Chinensis Cortex*, has shown substantial efficacy in treating various skin diseases, such as psoriasis, dermatitis, and cutaneous pruritus.

#### 3.3.4 Treatment of gout and arthritis

SGR is recognized for its ability to clear turbid dampness within the body and has been used to clinically address pain and inflammation associated with damp-heat, notably in conditions such as gout and arthritis. Integrating an SGR-based formula with conventional Western gout arthritis treatments has yielded enhanced outcomes. In a study by Yang, the addition of a decoction containing SGR, *Angelicae Pubescentis Radix*, Taxilli Herba, *Eucommiae Cortex*, and *Achyranthis Bidentatae Radix* to a standard Western pharmacological regimen for ankylosing spondylitis over a three-month period resulted in an 85.71 % effective rate, significantly surpassing the 46.67 % rate achieved with Western medicine alone [77].

Moreover, the use of SGR as a solo decoction in conjunction with etoricoxib tablets demonstrated increased efficacy compared to the administration of etoricoxib alone for patients with acute gouty arthritis characterized by damp-heat accumulation [78]. Clinically, high doses of SGR (30–60 g) have been prescribed as the primary treatment during arthritic flare-ups, with three to five doses typically sufficient to mitigate symptoms such as joint redness, swelling, heat, and pain [79].

#### 3.3.5 Other clinical applications and related proprietary Chinese medicine introduction

Beyond its primary clinical uses, SGR is implicated in a broad spectrum of therapeutic effects. Evidence from clinical trials suggests the efficacy of Fu Ling, a component associated with SGR, in the treatment of syphilis [80], phlegm-stasis migraine [81], and as an adjunctive to Western pharmaceuticals in managing hepatitis B and related acute and chronic liver failure [82]. To enhance patient compliance and convenience, several oral formulations incorporating SGR have been developed. These include the compound SGR granule for hyperuricemia by Guangzhou General Hospital of the Guangzhou Military Region, an SGR and Dandelion concoction for pelvic inflammatory disease by Xiangyu Pharmaceutical Co. Ltd., an oral SGR total glycoside preparation by Guangzhou Baiyunshan Jingxiu Tang, the Myriad pills blending SGR with Sarsaparilla and Dioscorea, as well as the previously discussed Gastric Health Combination and Jian Spleen and Skin Beauty Dispersion.

Topical applications also exist, such as the Wang Huo Huang lotion. SGR possesses a sweet, light, and gentle profile, contributing to a wide pharmacological applicability with minimal contraindications, thus lending itself to diverse pharmaceutical formulations. The continued development of SGR-related Chinese medicines is expected to expand their therapeutic reach to additional pathologies, with a particular emphasis on oral preparations to enhance therapeutic outcomes. Table 2 collates the varied clinical applications of SGR.

**Table 2:** Clinical applications of SGR.

Clinical applications	Pharmacological Models	Effects	Dosage	Pharmacodynamic constituents	references
Treatment of urinary system diseases	Patients with rheumatism	Detoxification and removing rheumatism	Include SGR: 30 g	SGR	[67]
	Patients with abnormal vaginal discharge	Treat female leucorrhea abnormalities	Include SGR: 30 g	Formula for removing dampness (e.g., SGR, Tsubaki Root and White Bark, Celosia, Coix Seed, and White Peony)	[68]
	Patients with nongonococcal urethritis	Treat non-gonococcal urethritis	Include SGR: 40 g	Tonglin Detoxification Tang (e.g., SGR, Herba Hedyotis, Lysimachia, Herba Houttuynaiae, and White fresh peel)	[69]
	Patients with chronic non-gonococcal prostatitis	Treat chronic non-gonococcal prostatitis significantly reduced the NIH–CPSI score, improved the quality of life and number of lecithin vesicles.	Include SGR: 25 g	Qingzhuo Tang (e.g., SGR, Peach kernel, safflower, Red Peony, and Salvia)	[70]
Treatment of digestive system diseases	Patients with helicobacter pylori infectious gastric ulcer	Treat Helicobacter pylori infectious gastric ulcer	Include SGR: 15 g	Antibacterial soup (SGR, Salvia, Forged corrugated, and Dai Ochre)	[71]
Treatment of skin diseases	Patients with psoriasis vulgaris (blood-heat type)	Reduce the levels of TNF-*α* and VEGF in the blood of patients with common psoriasis	Include SGR: 30 g	Indigo Naturalis decoction (e.g., SGR, Indigo Naturalis, Honeysuckle, Radix Glycyrrhizae, and Smilax China)	[74]
	Patients with psoriasis vulgaris of blood-hot	Treat psorasis vulgaris of blood-hot	Include SGR: 30 g	Jiawei Xiaoyin decoction (e.g., SGR, Buffalo horns, Dried Rehmannia root, Radix Paeoniae Rubra, and Ramuli Euonymi)	[75]
	Children with eczema	Treat infantile eczema with spleen deficiency	Include SGR: 5 g	Spleen and beautifying the skin powder (e.g., SGR, Astragalus mongholicus, Rhizoma Polygonati, and Rhizoma Atractylodis)	[76]
Treatment of gout and arthritis	Patients with ankylosing spondylitis	Treat ankylosing spondylitis	Include SGR: 30 g	Angelicae pubescentis and Loranthi decoction (e.g., SGR, Radix Angelicae pubescentis, Herba Taxilli, Cortex Eucommiae, and Radix Achyranthis Bidentatae)	[77]
	Patients with acute gouty arthritis	Treat acute gouty arthritis	SGR: 100 g	SGR mono-decoction combined with etoricoxib tablets	[78]
Other clinical applications	Patients with syphilis	Treat syphilis	SGR: 60 g	SGR combined with penicillin	[80]
	Patients with phlegm-stasis migrain	Treat phlegm-stasis migraine	Include SGR: 50g	SGR soup	[81]
	Patients with hepatitis B virus plus acute and chronic liver failure	Treat hepatitis B virus plus acute and chronic liver failure	Include SGR: 100g	SGR composition	[82]

#### 3.3.6 Safety assessment of SGR

An exhaustive review of historical and contemporary texts was undertaken to ascertain the safety profile and potential toxicity of SGR. This investigation, encompassing a range of pharmacopeias and materia medicas from various dynastic periods such as the Bencao Tujing by Su Song in the Song Dynasty, to the Bencao Yidu by Shen Mu in the Qing Dynasty, consistently reported an absence of adverse reactions associated with SGR. This lack of reported toxicity is in alignment with its classification as an edible substance and supports the herbal consensus regarding its safety. Seven monographs unequivocally classify SGR as non-toxic. Furthermore, the 2020 edition of the Chinese Pharmacopoeia describes SGR as “sweet, light, and flat,” and its affinity for the liver and stomach meridians suggests a non-irritant nature, corroborating its mildness. Acute toxicity assays in murine models have further substantiated the non-toxicity of SGR [83], offering evidence for its safe medicinal application and consumption.

Nevertheless, the Chinese Pharmacopoeia stops short of quantitatively defining SGR's toxicity, signaling residual uncertainty concerning its unequivocal safety. This gap denotes a necessity for pharmacological investigations to delineate standard safety parameters and toxicity thresholds.

Presently, there is no consensus standard for SGR's safety and toxicity levels. However, empirical data on customary dosages indicate a broad therapeutic window and an established safety margin. The conventional dosage spectrum for SGR extends from 15 to 60 g. For certain conditions, such as during acute episodes, dosages can be escalated to 240 g without reported adverse effects within the documented range of 9–240 g [84]. It is worth noting, however, that excessive dose increments beyond the established therapeutic range could potentially elicit gastrointestinal discomfort due to the organism's challenge in processing large quantities of the substance acutely. Such escalation would diverge from standard clinical practice and might not provide an accurate reflection of its safety profile at therapeutic doses.

Despite the high safety profile of SGR and its recognized role as an antidote to certain toxicities, its application is not without contraindications. Historical texts, including seminal works such as the Compendium of Materia Medica, advise against the concurrent use of SGR with tea, as well as with specific substances and conditions that provoke diaphoresis for releasing exterior pathogens or that stimulate the movement of qi. Furthermore, the Materia Medica specifies that SGR should not be taken with alcohol, various meats like those of cattle, sheep, chicken, and geese, nor in any context that encourages perspiration for expelling exterior pathogenic factors or mobilizing qi.

The Bencao Dongquan [85] cautions against the use of SGR in heat-clearing contexts, postulating that prolonged administration may precipitate heat deficiency and a consequent depletion of qi. This depletion, in turn, could result in internal dampness and stagnation. While no explicit prohibitions on SGR usage exist in contemporary pharmacopeias, prudent consideration of a patient's overall health, the method of decoction, and dietary habits is imperative.

Presently, the Chinese Pharmacopoeia lacks specific guidance on potential interactions and contraindications involving SGR. Ensuring the clinical safety of SGR necessitates a pharmacological inquiry into historical formulations bearing analogous medicinal properties. Such investigation could illuminate potential interactions and contraindications, thereby optimizing the therapeutic application of SGR.

### 3.4 Predictive analysis of the Q-markers of SGR

The integrity of Traditional Chinese Medicine (TCM) is paramount, serving as the cornerstone for the advancement and global adoption of the TCM industry. Numerous factors influence the quality of Chinese medicinal products, including species variation, the ecological parameters of cultivation, harvesting and post-harvest processing, manufacturing techniques, logistical considerations for transportation and storage, as well as the methodologies of extraction and purification. Additionally, the administration pathways of the drugs, the synergy and interplay among constituent compounds, and the specificity of prescriber formulations are critical.

The heterogeneity of quality in the Chinese medicinal products marketplace, the transition from regional to national standards, the empirical rigor of the Chinese Pharmacopoeia benchmarks, and the precision of quality control measures, all exemplify the multifaceted challenges associated with ensuring quality, standardization, and regulation of TCM. The task of ensuring safe usage is further compounded by the absence of comprehensive standardization and oversight in their clinical application.

Liu et al. have introduced the concept of Q-markers for Chinese medicinal products, advocating these markers as indicative of the safety and efficacy of such medicines, and signaling a novel direction for their quality assurance [86]. Research within the field of natural medicinal chemistry has identified several focal points concerning Chinese medicines: their intricate structural diversity, the specificity of compound types to certain taxa, their significant biological activities, and medicinal utility. The chemical biology underpinning these medicines and their affinity originates from the biogenic pathways responsible for the synthesis of these components, often localized to distinct tissues, organs, or cells at certain developmental stages.

The correlation between plant secondary metabolites and the quality and quantity of these compounds is determined by their biosynthetic pathways, extending to the specific species, subspecies, or cultivars. A thorough analysis into the biosynthesis and accumulation patterns of secondary plant metabolites has revealed that a multitude of factors, including the sample's origin, individual variation, strain, age, geographic and temporal collection specifics, and storage duration, influence these compounds' biosynthesis and accumulation [86]. While this complexity presents significant challenges in quantifying secondary metabolites, it concurrently highlights the sensitivity of their measurement.

Liu et al. have delineated foundational criteria for Q-markers, which include: (1) compounds intrinsically present in botanical drugs or those emerging during their processing, and (2) compounds integral to the botanical drugs' functionality, possessing definitive chemical structures. Furthermore, Bing et al. have posited that Q-marker identification should adhere to five key principles: efficacy, specificity, transferability and traceability, measurability, and suitability for prescription [87].

Liu et al. also proposed that several factors impinge upon the determination of TCM Q-markers, including the cellular and tissue specificity, organ-specific biosynthesis, developmental stage specificity, external growth conditions of botanical drugs, and standardized decoction or proprietary medicine preparation influences.

Given the widespread occurrence of SGR in China and its quality variation due to environmental, growth, and harvesting conditions, there is disparity in the quality of SGR decoctions available in the market. Predictive analysis for the Q-markers of SGR should, therefore, be premised on aspects such as chemical composition distinctiveness, clinical effectiveness, traditional medicinal roles, intrinsic medicinal attributes, and quantifiable chemical constituents.

#### 3.4.1 Q-marker prediction analysis of SGR based on plant affinities and evidence of chemical composition specificity

Globally, the Smilax genus encompasses approximately 200 species, distributed across tropical, subtropical, East Asian, and North American regions. Within China, 79 species have been identified, predominantly inhabiting locales south of the Yangtze River. Studies exploring the potential correlation between the germplasm and geographic distribution of Smilacina racemosa members have yielded inconclusive integrations of results. Zhang et al. investigated Smilax microdonta through CDDP analysis, revealing a substantial genetic diversity with 229 loci amplified, 98.25 % of which were polymorphic, thus demonstrating significant genetic variability within the Smilax genus [88]. This diversity was similarly echoed in findings utilizing RAPD markers. Research has also indicated a close genetic affinity between Smilacina racemosa and various Smilax species, including Smilax China and SGR. Chemical analysis focusing on the photosensitive constituents of SGR has revealed a discernible correlation between the concentration of each component and the plant's age [89]. This suggests that growth years are a determining factor in the chemical profile of SGR, which is a critical consideration for the prediction and identification of Q-markers in the species.

In their construction of chemical component-target (C-T) networks, Yong et al. discovered that flavonoids presented the highest target engagement with 287 targets, followed by stilbenes with 157, and organic acids at 74, along with steroids and saponins [90]. The pharmacodynamic action of a compound is generally believed to be a function of its interaction with molecular targets, with a higher number of targets often suggesting a more potent pharmacological effect. Consequently, flavonoids, stilbenes, organic acids, steroids, and saponins are posited as the primary active constituents underpinning the pharmacological efficacy of SGR.

Yan et al. isolated the active compound astilbin in SGR samples from five different regions, finding significant variance in astilbin levels across various collection periods [91]. As the artificial cultivation area of SGR has expanded to meet growing market demand, genetic diversity within its germplasm has increased, influencing the chemical composition of SGR's active ingredients due to genetic polymorphisms and environmental factors.

**Fig. 8:**
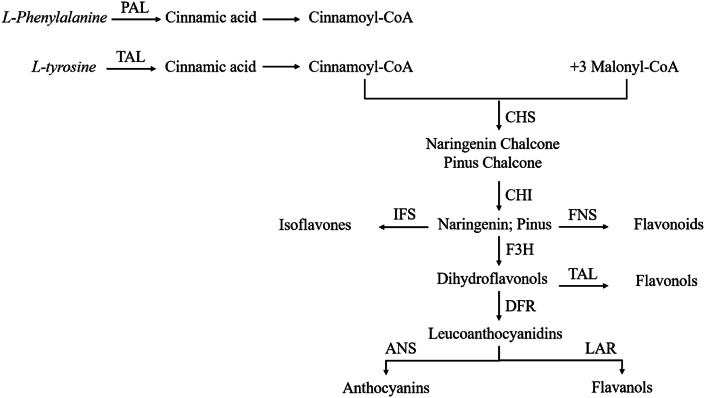
Biosynthesis pathway of flavonoid in SGR.

The biosynthesis of plant flavonoids initiates with the conversion of phenylalanine and tyrosine into coumaroyl-CoA via the phenylpropane pathway. This is followed by the conjunction with three molecules of malonyl-CoA to yield chalcone, which undergoes intramolecular cyclization to release dihydroflavonoids. These intermediates then diverge into various branches, synthesizing isoflavones, flavones, flavonols, anthocyanins, and flavanols [92]. The dihydroflavonol astilbin is notably indicative of the biological specificity of SGR, providing a foundation for Q-marker exploration. Based on this synthesis pathway, flavonoids are proposed as viable Q-markers for SGR. The biosynthetic routes of SGR flavonoids are elucidated in Fig. 8.

#### 3.4.2 Q-markers prediction analysis of SGR based on clinical efficacy

The clinical utility of SGR is well-documented, with contemporary pharmacological research elucidating the correlations between its chemical constituents and therapeutic applications. Evaluating the pharmacological bases of these effects has identified specific compounds as potential Q-markers. Although studies in natural medicinal chemistry have advanced our understanding of the diverse biological activities of individual monomers, their activities within botanical drugs can vary significantly.

Correlating the therapeutic attributes of SGR with the observed biological activities of various monomers yields notable insights. Primarily, astilbin in SGR exhibits antitumor, anti-inflammatory, and immunosuppressive properties [23,54]. It has been demonstrated to extend the latency of pain response in mice on a hot plate test. The crude flavonoid fraction from SGR has shown hepatoprotective effects against damage from cotton phenol [93]. Additionally, SGR's glycoproteins possess antiviral capacities, while smilaxin is known for its anti-stress ulcerative effects [23]. Smiglabrin, a component of SGR, has cardioprotective actions, ameliorating myocardial ischemia-reperfusion injury, and shows efficacy against *β*-adrenergic blockade and cerebral ischemia in mice [2]. A synergistic effect of astilbin with polysaccharides was observed in the modulation of macrophage-mediated inflammation [11]. Furthermore, the steroidal saponins in SGR are recognized for their anti-atherosclerotic and antithrombotic impacts [94], and its total saponins have shown selectivity towards sarcoma S180 cells.

However, not all chemical components are suitable as Q-markers—especially those that are ubiquitous or whose structures pose significant challenges in determination. It is thus posited that flavonoids (specifically astilbin), glycoproteins, and saponins (notably smiglabrin) can serve as reliable indicators for Q-marker identification aligned with the distinct clinical effects of SGR.

#### 3.4.3 Q-markers prediction analysis of SGR based on traditional potency

Traditional potency constitutes a cornerstone for the clinical utility of TCM, succinctly encapsulating the therapeutic attributes of these treatments. The extant Chinese Pharmacopoeia delineates the curative advantages of SGR, noting its capabilities in detoxification, moisture elimination, and the facilitation of joint collateral circulation. Historical texts, including the "Supplement to Materia Medica," "Southern Yunnan Materia Medica," "Compendium of Materia Medica," and "Materia Medica Seeking Truth," document SGR's pesticidal properties.

Contemporary pharmacological investigations corroborate SGR's traditional use in managing gout and arthritis—attributable largely to the xanthine oxidase inhibition by astilbin and related compounds [3,23,54,60]. The treatment efficacy of SGR for conditions such as gonorrhea, leukorrhea, and syphilis primarily arises from the anti-inflammatory and antibacterial actions of its flavonoid components [42,52,54]; however, flavonoids are deemed unsuitable as Q-markers due to their widespread presence.

SGR's capacity for detoxifying mercury powder and mitigating the toxicity of heavy metals like silver is predominantly reflected in the detoxifying properties of its constituents, including astilbin, resveratrol, astragaloside, and caffeic acid [67]. Based on these findings, it is reasonable to propose further exploration of astilbin and astragaloside as Q-markers for SGR. The therapeutic basis of SGR's traditional uses offers a significant frame of reference for the screening and identification of Q-markers.

#### 3.4.4 Q-markers prediction analysis based on the traditional medicinal properties of SGR

TCM possesses distinctive medicinal properties, and the associated organ tropism plays a role in clinical treatment and drug formulation, serving as potential indicators for Q-markers screening. According to the 2020 edition of the Chinese Pharmacopoeia, SGR is characterized by a neutral therapeutic impact, a sweet and mild flavor profile, and an affinity for the liver and stomach channels.

Incorporating TCM theory, the underlying material basis of SGR's sweet taste should reflect the inherent characteristics and functional implications of this flavor. Chemical analysis reveals that Chinese medicines with a sweet palate often comprise sugars, saponins, proteins, sterols, and lipids. These components exhibit antiviral, antibacterial properties, and possess the capability to modulate blood pressure, reduce blood sugar levels, and induce diuretic effects. Sweet-tasting botanical drugs are typically prescribed in conjunction with those possessing lighter tastes to promote diuresis and dispel dampness [23].

SGR constituents such as phenylpropanoids, steroids, and organic acids are secondary in significance to flavonoids and sugars concerning their contribution to the plant's medicinal profile. The pharmacological activities of SGR correspond with the outcomes of the medicinally focused chemical composition research. The investigations referenced herein suggest that polysaccharides, saponins, and sterols contribute significantly to the sweet flavor of SGR. Aligning with the fundamental criteria for quality markers, it is posited that the saponins and steroids in SGR may serve as suitable candidates for Q-markers screening.

#### 3.4.5 Q-markers prediction analysis of SGR based on the measurability of chemical components

Chemical component measurability informs the screening of Q-markers, as delineated by the determination methods and threshold requirements for astilbin in the 2020 Chinese Pharmacopoeia. Astilbin levels serve as indicators of the total flavonoid content in SGR, although the presence of isomers compromises the precision of such measurements, and thus a solitary analyte fails to comprehensively represent SGR quality. Predominantly, studies have utilized HPLC for quantifying astilbin and its isomers, along with astragaloside, 5-O-caffeoyl shikimic acid, and (−)-epicatechin. Chen et al. employed astilbin as an internal standard to concurrently evaluate astilbin and astragaloside levels in SGR by a one-test-multiple-evaluation technique [95], finding astilbin to be the most prevalent (0.5812%–4.897 %), followed by 5-O-caffeoyl shikimic acid (0.1063%–2.692 %), and engeletin (0.0312%–0.6771 %). Zhang et al. developed an HPLC fingerprint for SGR and advanced a method to simultaneously measure astilbin and resveratrol levels [96], uncovering significant variations among the compounds across samples. Xu et al. applied HPLC-DAD to simultaneously assess the content of (−)-epicatechin, 5-O-caffeoyl shikimic acid, astilbin, and its isomers in 84 SGR samples, with results ranging from trace amounts to 27.08 mg g^-1 [97]. Additionally, Sun et al. employed HPLC to quantify ferulic acid, astilbin, and resveratrol in SGR [98]. The quantification of ferulic acid, astilbin, and resveratrol in SGR samples yielded concentrations of 0.05964, 0.01455, and 0.01839 mg g^-1, respectively. Variations in pretreatment and drying processes influence the principal chemical constituents of SGR. Boiling and steaming as pretreatment methods resulted in increased levels of neoastilbin, neoisoastilbin, and isoastilbin. Contrarily, the concentration of astilbin, a designated Q-marker for SGR, remained constant or exhibited marginal reduction when compared to untreated samples [99]. In contrast, the bioavailability study by Zheng et al. highlighted negligible absorption of neoastilbin and astilbin in rats, with no marked difference noted [100]. These methodologies contribute to the standardization of SGR quality control. Nonetheless, the establishment of precise quantification techniques for specific, measurable chemical components is imperative to enhance the quality control and assessment of SGR. The consistently quantified flavonoids, notably astilbin and astragaloside, along with phenolic glycosides such as resveratrol, should be considered as critical reference parameters for SGR's Q-markers.

**Fig. 9:**
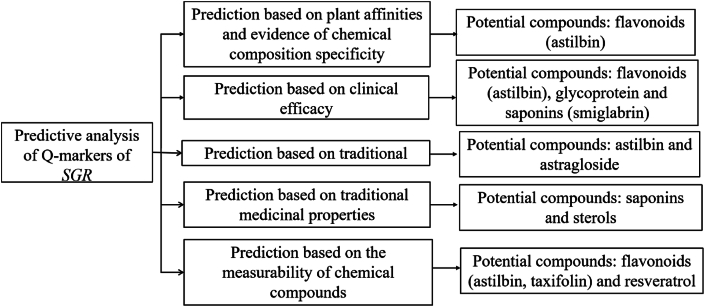
Potential Q-markers of SGR.

Based on plant phylogenetic relationships, chemical profiles, clinical effectiveness, historical medicinal use, and chemical component quantifiability, the Q-markers for SGR are projected to encompass astilbin, glycoproteins, astragaloside, saponins, and sterols, as depicted in Fig. 9. Among these, astilbin is identified as having the highest potential due to its fulfillment of criteria across plant affinity, chemical specificity, clinical efficacy, and quantifiability. However, due to isomeric interference in accurately quantifying astilbin, concurrent assessment of other constituents—such as resveratrol, ferulic acid, astragaloside, (−)-epicatechin, neoastilbin, neoisoastilbin, and isoastilbin—is required for more robust results. In the current quality control framework of Traditional Chinese Medicine (TCM), the oversight of TCM preparations is hampered by deficiencies, including the absence of comprehensive quality controls from raw materials to finished products, inadequate correlation between process parameters, product quality, production equipment, and the inefficiency of the traditional industry alongside low levels of equipment automation. The implementation of a complete chain quality control approach, anchored by Q-markers, has the potential to enhance precision in every operational phase of production and preparation, thereby aligning more closely with the stringent requirements for drug safety, efficacy, and quality management. Despite the comprehensive perspective and central relevance of Q-marker screening for quality assurance, the method's practical complexity resides in the initial Q-marker pre-screening phase.

### 3.5 Q-markers validation analysis based on network pharmacology and molecular docking

#### 3.5.1 Construction of SGR-Q-markers-targets network

Subsequent to our analysis, we posited that components such as astilbin, astragaloside, resveratrol, diosgenin, neoastilbin, neoisoastilbin, stigmasterol, and *β*-sitosterol could serve as Q-Markers for SGR. These were selected on the basis of their Oral Bioavailability (OB) being greater than or equal to 30 % and their Drug-Likeness (DL) score being greater than or equal to 0.18, as per the TCMSP database (http://tcmspw.com) [101]. Upon entering the SMILES data of these 12 Q-Markers into the Swiss Target Prediction database, duplicate targets were excised, yielding a total of 305 distinct targets [102].

**Fig. 10:**
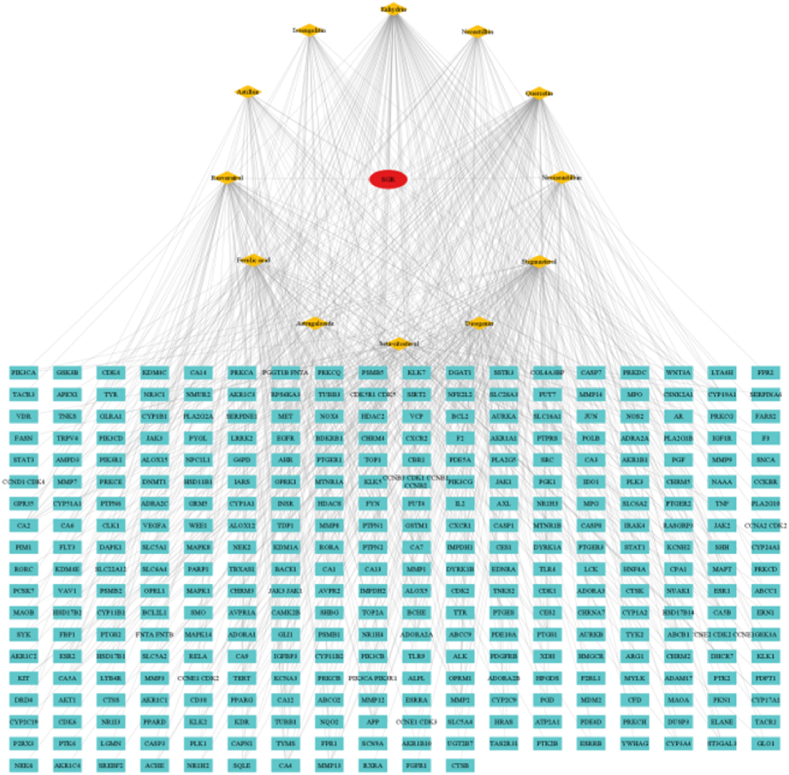
SGR-Q-Markers-targets interaction network. Red oval represents SGR, yellow diamond represents Q-markers, and sky blue rectangle represents targets. (For interpretation of the references to colour in this figure legend, the reader is referred to the Web version of this article.)

Utilizing Cytoscape 3.9.1, an interaction network between SGR-Q-Markers and their targets was constructed, as displayed in Fig. 10. This network consists of 348 nodes and 693 edges. Network topology analysis revealed that chemical components are often associated with multiple gene targets, which in turn may correspond to several chemical components simultaneously. Detailed results are documented in Table 3.

**Table 3:** List of Q-markers of SGR.

Mol	Compound name	OB/%	DL	targets
MOL013119	Enhydrin	40.56	0.74	100
MOL000098	Quercetin	46.43	0.28	100
MOL000449	Stigmasterol	43.83	0.76	97
MOL012744	Resveratrol	19.07	0.11	69
MOL000360	Ferulic acid	39.56	0.06	60
MOL000358	Beta-sitosterol	36.91	0.75	43
MOL000546	Diosgenin	80.88	0.81	43
MOL013118	Neoastilbin	40.54	0.74	39
MOL004575	Neoisoastilbin	36.46	0.74	39
MOL004575	Astilbin	36.46	0.74	36
MOL004567	Isoengelitin	34.65	0.70	34
MOL000401	Astragaloside	46.79	0.11	21

#### 3.5.2 Construction of SGR-Q-markers-targets network

**Fig. 11:**
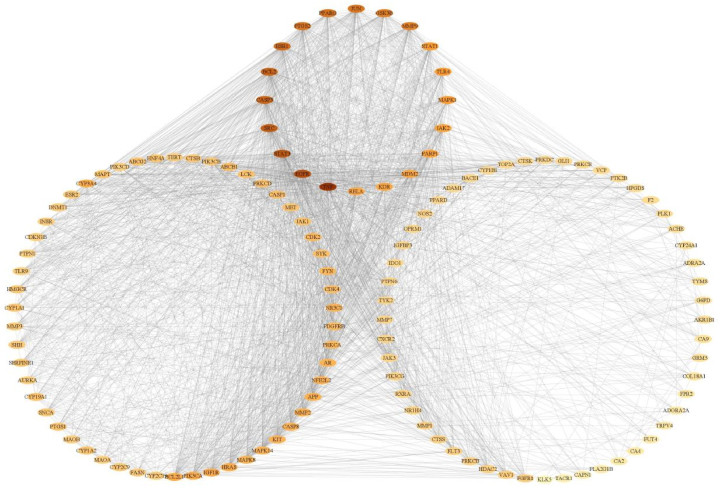
PPI network of Q-Marker related targets in *SGR*.

Employing the STRING database, an initial protein interaction network was assembled for the 305 targets, as represented in Fig. 11. Here, nodes symbolize proteins, and edges denote the interactions amongst them. A higher degree of network connectivity indicates stronger protein associations. After importing the STRING database files into Cytoscape 3.9.1, the CytoNCA plug-in was applied to conduct network topology analysis and to identify pivotal nodes. The proteins with the highest Degree values include TNF (Degree = 143), EGFR (Degree = 124), STAT3 (Degree = 118), SRC (Degree = 114), CASP3 (Degree = 109), BCL2 (Degree = 109), ESR1 (Degree = 105), PTGS2 (Degree = 99), PPARG (Degree = 99), and JUN (Degree = 98).

#### 3.5.3 Molecular docking between Q-markers and core targets

**Fig. 12:**
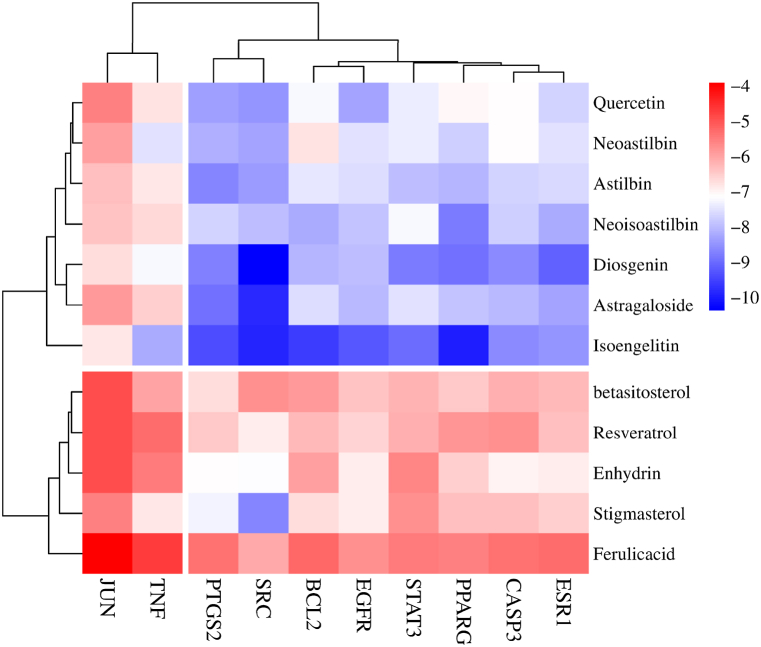
The docking thermogram of Q-markers and core proteins.

Q-Markers underwent docking with core targets. The three-dimensional structures of the core targets were obtained from the Protein Data Bank (pdb) database, and the mol2 structures of the corresponding Q-Markers were retrieved from the TCMSP database. These were then converted to pdb format and imported into AutoDock Vina for docking [103]. The molecular docking outcomes, depicted in Fig. 12, reveal binding energies below −4.5 kcal mol^-1, signifying favorable binding affinities between the projected core targets and key Q-Markers.

## 4 Conclusions

The investigation into SGR, a traditional botanical drug with dual roles as a food and therapeutic entity, presents significant research and development prospects. There has been a notable increase in the extraction and identification of SGR's chemical constituents, particularly flavonoids, which are garnering attention across various research domains. It is imperative to extend the exploration of SGR's functional and active monomeric compounds utilizing contemporary research techniques within interdisciplinary and multilevel frameworks, to elucidate the structure-activity relationship inherent to Chinese medicine, characterized by its multifaceted components and targets. Throughout history, from medieval times to the contemporary era, SGR has been widely applied in clinical treatments; yet, the surge in artificial cultivation has not been paralleled by rigorous quality control studies and the formulation of a standardized quality benchmark. Consequently, establishing robust and efficacious quality evaluation protocols is crucial for SGR's quality assurance.

In this study, we have adopted the novel concept of Q-markers to forecast and scrutinize the Q-markers of SGR, with the aim of furnishing reference perspectives and methodologies for the management and selection of SGR's Q-markers. Given SGR's application in treating an array of diseases, including cancer, the creation of a cogent quality control system is pivotal for fostering its development and clinical use.

It must be underscored, however, that the bulk of the pharmacological research pertaining to SGR's efficacy is presently confined to cellular and animal models. The lack of clinical trials investigating the effects of SGR in human subjects limits our understanding of its potential anti-inflammatory, analgesic, immunomodulatory, antitumor, anti-atherosclerotic, and expectorant properties. The current edition of the Chinese Pharmacopoeia notes SGR's utilization for conditions such as syphilis, mercury poisoning, limb contractures, tendon discomfort, damp-heat gonorrhea, various gynecological disorders, carbuncles, cervical lymphatic tuberculosis, and scabies. While contemporary pharmacological studies have illuminated SGR's activity, discrepancies between animal and human physiology, as well as between in vivo and in vitro environments, necessitate further clinical validation to ascertain its pharmacological activities in humans.

In summary, this investigation has synthesized SGR's quality attributes across multiple dimensions, conjectured its Q-markers, and authenticated these via network pharmacology and molecular docking, providing a foundational reference for the holistic control of decoction piece quality.

## Funding

This research did not receive any specific grant from funding agencies in the public, commercial, or not-for-profit sectors.

## Data availability statement

Date will be made available on request.

## CRediT authorship contribution statement

**Mingxin Guo:** Writing – review & editing, Writing – original draft. **Jiaqi Zeng:** Writing – review & editing, Writing – original draft, Validation. **Wang Zhanle:** Writing – review & editing, Conceptualization. **Shen Ying:** Writing – review & editing, Supervision, Conceptualization.

## Declaration of competing interest

The authors declare that they have no known competing financial interests or personal relationships that could have appeared to influence the work reported in this paper.

## Abbreviations

TCM: Traditional Chinese Medicine
SGR: *Smilacis Glabrae Rhizoma*
Q-markers: quality markers
CNKI: Chinese National Knowledge Infrastructure
DDPH: 1,1-Diphenyl-2-picrylhydrazyl
ABTS: Diammonium 2,2'-azino-bis(3-ethylbenzothiazoline-6-sulfonate)
IL: interleukin
NO: nitric oxide
NF-κB: nuclear factor kappa-B
ERK: extracellular regulated protein kinases
c-JNK: c-Jun N-terminal kinase
ROS: reactive oxygen species
TNF: tumor necrosis factor
LPS: lipopolysaccharide
IFN-*α*: Interferon-*α*
VEGF: Vascular endothelial growth factor
CDDP: Compound Danshen Dripping Pills
RAPD: Random Amplification Polymorphic DNA
PAL：: Phenylalaninammo-nialyase
TAL: Tyrosineammonialyase
CHS: Chalcone synthase
CHI: Chalconeisomerase
IFS: Isoflavonesynthase
FNS: Flavonol synthase
F3H: Flavanone 3-*β*-hydroxyalse
DFR: Dihydroflavonol 4-reductase
ANS: Anthocyanin synthetase
LAR: Leucoanthocyanidin reductase
